# Rates of hospitalization and death for all-cause and rotavirus acute gastroenteritis before rotavirus vaccine introduction in Kenya, 2010–2013

**DOI:** 10.1186/s12879-018-3615-6

**Published:** 2019-01-11

**Authors:** Richard Omore, Sammy Khagayi, Billy Ogwel, Reuben Onkoba, John B. Ochieng, Jane Juma, Stephen Munga, Collins Tabu, Sergon Kibet, J. Pekka Nuorti, Frank Odhiambo, Jason M. Mwenda, Robert F. Breiman, Umesh D. Parashar, Jacqueline E. Tate

**Affiliations:** 1 Kenya Medical Research Institute, Center for Global Health Research (KEMRI-CGHR), Kisumu, Kenya; 2 Health Sciences Unit, Faculty of Social Sciences, University of Tampere, Tampere, Finland; 3 Division of Disease Surveillance and Response, Ministry of Public Health and Sanitation, Nairobi, Kenya; 4 WHO Country Office for Kenya, Nairobi, Kenya; 5 WHO Regional Office for Africa (WHO/AFRO), Brazzaville, Congo; 6 Division of Viral Diseases, US Centers for Disease Control and Prevention, Atlanta, GA USA; 7 Global Health Institute, Emory University, Atlanta, GA USA

**Keywords:** Rotavirus, Morbidity, Mortality, Children, Kenyan

## Abstract

**Background:**

Rotavirus vaccine was introduced in Kenya immunization program in July 2014. Pre-vaccine disease burden estimates are important for assessing vaccine impact.

**Methods:**

Children with acute gastroenteritis (AGE) (≥3 loose stools and/or ≥ 1 episode of unexplained vomiting followed by loose stool within a 24-h period), hospitalized in Siaya County Referral Hospital (SCRH) from January 2010 through December 2013 were enrolled. Stool specimens were tested for rotavirus (RV) using an enzyme immunoassay (EIA). Hospitalization rates were calculated using person-years of observation (PYO) from the Health Demographic Surveillance System (HDSS) as a denominator, while adjusting for healthcare utilization at household level and proportion of stool specimen collected from patients who met the case definition at the surveillance hospital. Mortality rates were calculated using PYO as the denominator and number of deaths estimated using total deaths in the HDSS, proportion of deaths attributed to diarrhoea by verbal autopsy (VA) and percent positive for rotavirus AGE (RVAGE) hospitalizations.

**Results:**

Of 7760 all-cause hospitalizations among children < 5 years of age, 3793 (49%) were included in the analysis. Of these, 21% (805) had AGE; RV was detected in 143 (26%) of 541 stools tested. Among children < 5 years, the estimated hospitalization rates per 100,000 PYO for AGE and RVAGE were 2413 and 429, respectively. Mortality rate associated with AGE and RVAGE were 176 and 45 per 100,000 PYO, respectively.

**Conclusion:**

AGE and RVAGE caused substantial health care burden (hospitalizations and deaths) before rotavirus vaccine introduction in Kenya.

## Background

Rotavirus is the most common cause of vaccine-preventable severe acute gastroenteritis (AGE) among infants and young children worldwide [1, 2]. In 2013, RVAGE was estimated to cause 215,000 global deaths among children < 5 years of whom 2% (~ 4000) were from Kenya [3] alone. In Kenya, RVAGE accounts for 19% (~ 9000) of annual hospitalizations among children < 5 years [4]. Two RV vaccines Rotarix® (GlaxoSmithKline), and RotaTeq® (Merck & Co.), are approved and recommended by the World Health Organization (WHO) for global use [5]. Efficacy and effectiveness studies of these vaccines have shown significant reduction in AGE and RVAGE associated hospitalizations and deaths among children < 5 years in both clinical trials and in settings where they have been incorporated into the national immunization programs [1, 6–10]. Consistent with data from Mexico [6] and Brazil [7, 11], African countries that were early introducers of RV vaccines including Malawi [10], Ghana [12], and Rwanda [13], have shown remarkable declines in childhood morbidity and mortality associated with AGE and RVAGE. Furthermore, the cost benefit of these vaccines has equally been demonstrated [4, 7, 14] .RV vaccine (Rotarix®) was introduced into the Kenya national immunization program in July 2014. Recent population-based data on pre-vaccine disease rates are not available in Kenya. However, such data are needed to evaluate the impact of vaccination program and may help county and national level governments, regional and global decision makers with evidence needed to support investment in these vaccines.

We examined baseline rates of AGE and RVAGE specific hospitalizations and deaths among children < 5 years in rural western Kenya from January 1, 2010 to December 31, 2013 before RV vaccine introduction in Kenya.

## Methods

### Study site

Rotavirus surveillance and the Health Demographic Surveillance System (HDSS) platform in our study setting has been detailed elsewhere [15, 16]. In brief, the HDSS site is located in Siaya County in rural western Kenya. The HDSS is a longitudinal study that monitors births, deaths, out and in-migrations and other demographics of a defined population [16]. Our study was conducted in Karemo HDSS area within Siaya county referral hospital (SCRH) — the main regional referral hospital in this setting.

### Rotavirus surveillance and laboratory methods

As part of the African-based, World Health Organization (WHO) coordinated RV rotavirus disease surveillance network [17], we conducted hospital-based prospective surveillance for RVAGE within the Kenya Medical Research Institute (KEMRI) operated HDSS area in Karemo [15]. Children aged 0–59 months residents of Karemo HDSS, hospitalized at the in-patient department of SCRH with AGE; defined as ≥3 looser than normal stools and/or ≥ 1 episode of unexplained vomiting followed by loose stool within a 24-h period beginning within the 7 days before seeking healthcare from January 1, 2010 to December 31, 2013 were eligible for enrolment. Trained health facility recorders approached all eligible patient children, explained the study and administered a questionnaire on demographics to their caretakers after obtaining informed consent. A study clinician then examined these patients and administered the standardized questionnaire to their parent/caretaker to gather information about symptoms, medical history, laboratory investigations, diagnosis, treatment and outcome of hospitalization. A whole stool specimen was collected from each participant in a plastic diaper from which at least 2 ml of stool was scooped into a specimen container using a sterile spatula within 48 h of admission, transported on the same day to the enteric laboratory based at the KEMRI-CGHR, and finally tested for rotavirus using a commercial enzyme immunoassay (EIA) (Rotaclone Kit, Meridian Bioscience). A case of RVAGE was defined as an AGE patient with a RV positive stool specimen.

### Data management

Details of the enrolment, testing and data management have been described previously [15]. In brief, we linked clinic data to laboratory results and to the longitudinal data including cause of death from verbal autopsy (VA) from the HDSS. During data collection, built-in software in the electronic questionnaire with built-in checks and controls ensured quality control. The linked data were then uploaded and managed using a Microsoft SQL Server 2008 database. Data were analysed using SAS version 9.4 (SAS Institute, Inc. Cary, North Carolina, USA).

### Statistical analysis

#### Descriptive analysis for AGE and RVAGE

Proportion of admissions due to AGE was calculated by dividing the number of AGE cases by the number of all-cause admissions at SCRH who were residents of Karemo HDSS during the study period. The proportion of admissions that were associated with RVAGE was calculated by dividing the number of RV positive stool samples with the total samples collected and tested. Positivity rates by month and patient characteristics (age, gender, clinical features and illness outcome) were calculated. These proportions were plotted by month to show seasonality.

#### Analysis of RVAGE, disease severity and risk factors

The severity of RVAGE was assessed by using the 20-point Vesikari score [18].A score of less than 11 was categorized as mild while a score of 11 or more was classified as severe. Bivariate comparison of the laboratory-confirmed RV positivity and patient characteristics and treatment outcomes were evaluated using chi-square tests.

#### Incidence rates of hospitalization and mortality due to AGE and RVAGE

We used person-years of observation (PYO) contributed by all children aged less than 5 years residents of Karemo region during the study period as the denominator. As described previously [15], we calculated PYO by totaling person-time for all children aged 0–59 months who met HDSS residency requirement during the 4-year study period from 1st January 2010 or date of enrolment (if after) until they exited or lost their HDSS residency status through out-migration or death.

The crude hospitalization rates were calculated by dividing the total number of AGE and RVAGE hospitalizations by the PYO contributed by children aged 0–59 months for the period that they met residency criteria for the HDSS.

We used two adjustments for the hospitalization rates. First, to account for possible missed AGE cases, we divided the crude rate of AGE and RVAGE by the proportion of all in-patients who met the stool collection criteria, whether a sample was collected or not.

The second adjustment accounted for children with AGE or possibly RVAGE who did not reach or attend a sentinel health care facility as reported from a population-based, healthcare utilization and attitude surveys (HUAS) for diarrhoea—a separate household survey conducted within the HDSS during the current RVAGE surveillance period [19].The HUAS revealed that the frequencies of seeking care for moderate-to-severe diarrhoea (MSD) from a hospital were 69, 70, 67, 57 and 64% for children aged 0–5, 6–11, 12–23, 24–59 and 0–59 months, respectively (GEMS-Kenya unpublished data). The 95% confidence intervals (CI) were calculated around crude rates by using the PEPI method [20]. Crude rates were then adjusted using Delta method [21, 22]. The adjusted hospitalization rates were finally stratified and reported by age groups that included; 0–5, 6–11, 12–23, 24–59, 0–11 and 0–59 months.

#### AGE and RVAGE mortality rates

Deaths were recorded at household level through regular interviews of HDSS residents. Diarrhoea as a cause of death was derived from Verbal autopsy (VA). The VA methodologies, coding and interpretation are described elsewhere [23, 24]. Upon the death of an HDSS resident, a trained village-based reporter sent a notification to HDSS data team. After a mourning period of at least 3 weeks, the interviewer from the HDSS approached the most appropriate interviewee who was closest to the deceased to administer a detailed questionnaire focusing on the signs, symptoms and medical history of the deceased. The VA data were collected electronically, validated and processed using an InterVA program, which is a probabilistic computer-based expert opinion algorithm that determines the most probable cause of death as described elsewhere [24]. We calculated the number of deaths attributed to RV by multiplying the total under-five deaths among HDSS residents in the study area by the proportion of deaths attributable to diarrhoea by VA, and the proportion of hospitalized AGE episodes attributable to RV in each of the various age groups as described below.

$$ No\  of\ deaths\ attributable\ to\  RV={\displaystyle \begin{array}{l}\left( Total\ under- five\ deaths\ among\ HDSS\ residents\ in\ study\ area\right)\\ {}{}^{\ast }\ \left( proportion\ of\ deaths\ attributable\ to\ diarrhea\right)\\ {}{}^{\ast }\ \left( proportion\ of\ hospitalized\  AGE\  episodes\ attributable\ to\  RV\right)\end{array}} $$

Mortality rates associated with rotavirus gastroenteritis were obtained by dividing the number of deaths attributed to rotavirus by the total PYO in each of the specific age groups as described above.

## Results

### Enrolment profile and patient characteristics

**Fig. 1:**
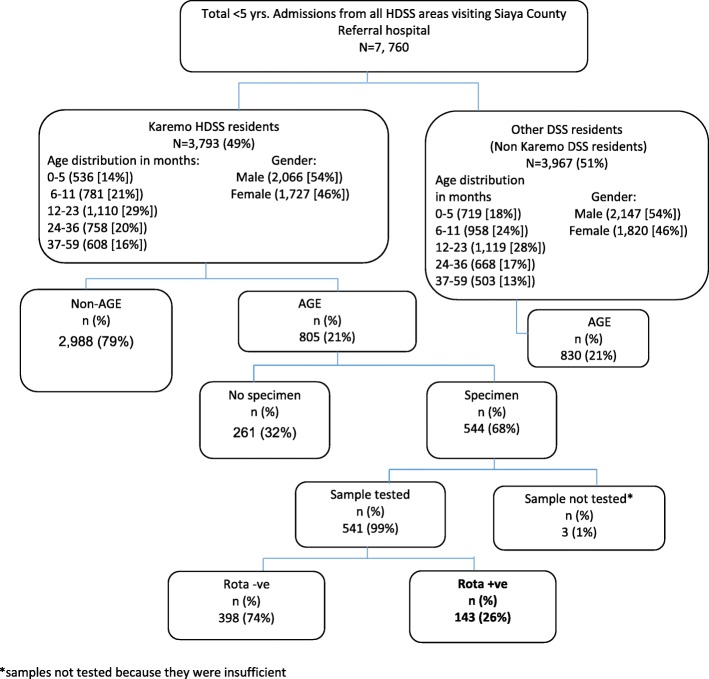
Flow diagram of Karemo DSS residents < 5 yrs. who were hospitalized and enrolled in the study from Siaya county referral hospital, western Kenya 2010–2013

During the study period, a total of 7760 all-cause hospitalizations among children < 5 years of age were recorded at the SCRH paediatric ward, out of which 3793 were Karemo HDSS resident population. Among the 3793 Karemo HDSS resident children, 805 (21, 95%CI: 20–23) children were hospitalized due to AGE (Fig. 1). RV-positivity among hospitalized children from Karemo with AGE was more pronounced in infants (< 12 months of age), then toddlers (12–23 months of age), and was least in school-age children (24–59 months of age) (Table 1). Characteristics of patients who had stool specimen**s** collected and those who did not have specimens collected are shown in Table 2.

**Table 1:** Characteristics of children< 5 years hospitalized at SCRH with all cause morbidity, AGE and RVAGE, 2010—2013

Characteristics	All Cause Hospitalizations from Karemo DSS (*N* = 3793)	AGE Hospitalizations (*N* = 805)	AGE specimen collected and tested for RV ҂ (*N* = 541)	Proportion tested RV Positive (*N* = 143)
	n	n (row %)	n (row %)	n (row %)
Sex				
Male	2066	461 (22%)	309 (67%)	87 (28%)
Female	3793	344 (20%)	232 (67%)	56 (24%)
Year				
2010	920	224 (24%)	147 (66%)	43 (29%)
2011	1335	313 (23%)	206 (66%)	53 (26%)
2012	822	147 (18%)	93 (63%)	26 (28%)
2013	716	121 (17%)	95 (79%)	21 (22%)
Age (Months)				
0–5	536	205 (38%)	141 (69%)	45 (32%)
6–11	781	285 (36%)	204(72%)	57 (28%)
12–23	1110	205 (18%)	138 (67%)	30 (22%)
24–36	687	70 (10%)	40 (57%)	9 (23%)
37–59	679	40 (6%)	18 (45%)	2 (11%)

҂ 3 samples collected were not tested

**Table 2:** Characteristics of Karemo resident children < 5 years hospitalized with AGE who had stool collected and those without stool collected, Siaya County Referral Hospital, Western Kenya, 2010–2013

Characteristic	Stool Sample Collected (*N* = 544)	Stool Sample Not collected (*N* = 261)	*P*-value
	n (%)	n (%)	
Age			
0–5 months	143 (26)	62 (24)	0.0034
6–11 months	204 (38)	81 (31)	
12–23 months	139 (26)	66 (25)	
24–59 months	58 (11)	52 (20)	
Gender			
Male	312 (57)	149 (57)	0.9
Female	232 (43)	112 (43)	
Vesikari Score			
Severe	268 (49)	93 (36)	< 0.0001
Mild	276 (51)	168 (64)	

Of the 541 stool samples collected, 204 (38%) were from infants aged 6–11 months. There was no difference in stool collection by gender. Furthermore, we did not observe any statistical difference in rotavirus positivity in male versus female patients among infants aged < 12 months ((69/211 [32.7%]) vs. (42/165 [25.4%]), OR = 1.42, *p* = 0.13),toddlers aged 12–23 months ((9/61 [14.7%]) vs. (12/46 [26.1%]), OR = 0.49, *p* = 0.15), or in children aged 24–59 months ((9/37 [24.3%]) vs. (2/21 [9.5%]), OR = 3.05, *p* = 0.18), respectively.

**Fig. 2:**
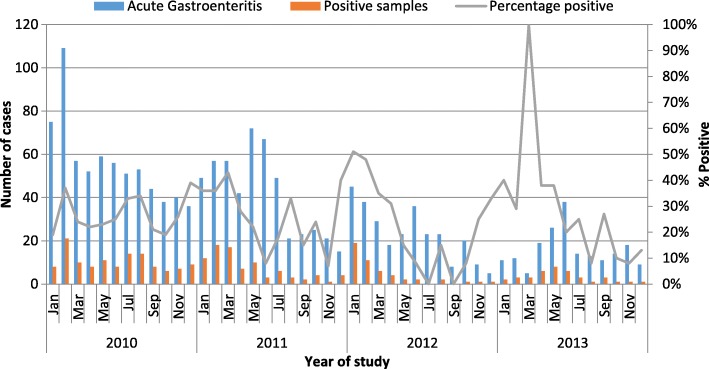
Rotavirus infection trends among hospitalized children < 5 years from Karemo DSS resident population seeking Care from Siaya County Referral Hospital, western Kenya 2010–2013

The overall annual proportion of rotavirus detection ranged from 43/147 (29.3%) in 2010 to 21/95 (22.1%) in 2013 and the annual proportion of samples detected with rotavirus did not differ significantly over the 4-year study period. Rotavirus hospitalizations were seen throughout the year over the surveillance period, but peaked from January through March and around August–September each year during study period (Fig. 2).

Compared with non-RVAGE cases, RVAGE cases were younger ((median age = 8 Interquartile range [IQR] 5–12) vs. 9 [IQR: 6–15] months; *p* < 0.032)), more likely to present with vomiting **(**(126/143 (88.1%) vs. 297/397 (74.8%)**),** and more likely to be classified as severe by Vesikari score (88/143 (61.5%) vs. 179/398 (44.9%), *p*-value = 0.0007).(Table 3).The length of hospitalization was similar for RVAGE compared to non-RVAGE (number of hospitalization days 4 [IQR] 3–6 vs. 4 [IQR] 3–6, *p*-value = 0.564).

**Table 3:** The Vesikari scores for severity of illness among RVAGE and non- RVAGE hospitalized children <5 yrs. in Siaya County Referral Hospital, western Kenya, 2010–2013

Variable	Points assigned	Rotavirus-positive	Rotavirus-negative	*P*-value
		*N* = 143	*N* = 398	
Duration of diarrhea (days)				
1–4	1	119 (83)	281 (70%)	0.0089
5	2	4 (3%)	31 (8%)	
> =6	3	20 (14%)	86 (22%)	
Max no. diarrhea/24 h				
1–3	1	41 (29%)	153 (38%)	0.1091
4–5	2	75 (52%)	177 (45%)	
> =6	3	27 (19)	68 (17%)	
Duration of vomiting (days)		*N* = 126	*N* = 296	
1	1	22 (15.4)	77 (19.4)	0.165
2	2	27 (18.9)	57 (14.4)	
> =3	3	77 (53.8)	162 (40.9)	
Max no. vomiting/24 h				
1	1	8 (7%)	39 (13%)	0.1003
2–4	2	95 (75%)	214 (72%)	
> =5	3	23 (18%)	43 (15%)	
Fever				
< 37.0	0	74 (52%)	196 (49%)	0.0435
37.1–38.4	1	58 (40%)	157 (40%)	
38.5–38.9	2	8 (6%)	12 (3%)	
> =39	3	3 (2%)	33 (8%)	
Dehydration		*N* = 55	*N* = 139	
1–5%	2	48 (87%)	117 (84%)	0.5852
> =6%	3	7 (13%)	22 (16%)	
Treatment*	2	143 (100%)	398 (100%)	–

*All cases were treated in the ward

### Hospitalization attributed to AGE in Karemo HDSS

The highest annual hospitalization rate (per 100,000 PYO) associated with AGE was observed in 2011 followed by 2010, 2012 and 2013 in descending order. The annual incidence (per 100,000) of hospitalizations due to all cause AGE was highest among infants and children aged 6–11 months remained most affected.

### Hospitalization attributed to RVAGE among children < 5 years from Karemo HDSS

Incidence rates of RVAGE associated hospitalization was highest among infants, particularly among those aged 6–11 months. We observed the highest RVAGE hospitalization rate in 2011 followed by 2010, 2012 and 2013 in decreasing order. Hospitalization rates for AGE and RVAGE are shown in Table 4.

**Table 4:** Adjusted† rates and 95% Confidence Intervals of hospitalization attributed to AGE and rotavirus per 100,000 Person-Years among in-patients aged 0–59 months residents of Karemo HDSS in Rural Western Kenya, 2010–2013

Year	Adjusted Rates^†^ of Hospitalization attributed to AGE						Adjusted Rates^†^ of Hospitalization attributed to RVAGE					
	0–5 months	6–11 months	12–23 months	24–59 months	0–11 months	0–59 months	0–5 months	6–11 months	12–23 months	24–59 months	0–11 months	0–59 months
2010	8612 (7854–9370)	8468(7717–9220)	7622(6909–8335)	619(416–822)	8478(7726–9230)	2718(2292–3144)	2153(2017–2289)	1540(1425–1654)	640(566–714)	44(25–64)	1804(1680–1928)	522(455–589)
2011	9428(8601–10,256)	12,317(11371–13,263)	12,900(11932–13,868)	927(667–1186)	10,913(10023–11,803)	3782(3258–4306)	1751(1627–1875)	2648(2495–2800)	664(588–741)	129(96–163)	2220(2080–2360)	640(565–716)
2012	5744(4918–6569)	5418(4617–6220)	4815(4599–5570)	365(157–573)	5526(4716–6335)	1745(1290–2200)	1336(1222–1449)	834(744–923)	352(148–410)	43(23–63)	1047(946–1148)	309(254–363)
2013	3604(2912–4296)	5335(4493–6178)	2916(2294–3539)	476(225–728)	4512(3737–5286)	1437(1000–1874)	772(703–841)	1307(1217–1396)	114(87–140)	22(10–33)	1055(974–1135)	249(210–289)
2010–2013	6806(6045–7568)	7798(6983–8614)	7010(6236–7783)	597(371–822)	7302(6513–8091)	2413(1959–2866)	1494(1383–1606)	1560(1446–1673)	439(379–499)	60(37–82)	1520(1408–1632)	429(369–488)

†Adjusted by applying the proportion of samples collected out of all the acute gastroenteritis admission in the hospital and health seeking behavior in the HDSS for children with reported diarrhoea at home

### Mortality attributed to AGE and RVAGE

Discharge information was available for 531 (98%) of the hospitalizations due to AGE of whom 33 (6.2%) died during hospitalization. The case-fatality proportion among RVAGE ((4.2%), [6/142]) compared to that observed from non-RVAGE ((6.9%), [27/389]) cases was similar, *p* = 0.26.

The highest mortality rates of AGE and RVAGE were observed among infants (< 12 months of age), and remained most elevated among infants aged 6–11 months. Annual mortality rates associated with RVAGE were stable between 2010 and 2011, but increased before RV vaccine introduction, especially among children aged 6–11 months. Mortality rates attributed to AGE and RVAGE are shown in Table 5.

**Table 5:** Rates and 95% Confidence Interval of deaths attributed to AGE and rotavirus per 100,000 Person-Years among in-patients aged 0–59 month’s residents of Karemo HDSS in Rural Western Kenya, 2010–2013

Mortality rates attributed to AGE per 100,000 Person-Years							Mortality rates attributed to Rotavirus per 100,000 Person-Years					
Year	0–5 months	6–11 months	12–23 months	24–59 months	0–11 months	0–59 months	0–5 months	6–11 months	12–23 months	24–59 months	0–11 months	0–59 months
2010	434(393–474)	475(432–517)	238(140–117)	100(80–120)	450(409–492)	172(146–198)	165(140–190)	119(97–140)	42(29–55)	33(22–44)	144(121–168)	46(33–59)
2011	342(306–378)	558(512–604)	353(316–389)	90(72–109)	452(411–494)	214(185–242)	99(80–119)	156(132–181)	63(48–79)	18(10–26)	131(109–154)	50(36–64)
2012	444(403–486)	314(280–349)	117(96–138)	43(30–56)	369(331–407)	118(97–140)	138(115–161)	91(72–110)	30(20–41)	3(0–6)	111(90–131)	56(41–70)
2013	368(331–406)	928(869–988)	225(195–254)	68(52–84)	649(599–699)	207(178–235)	92(73–111)	297(263–331)	25(15–34)	13(7–20)	188(161–215)	33(22–44)
2010–2013	401(362–441)	558(511–604)	210(181–238)	75(58–92)	479(436–521)	176(150–202)	124(103–146)	156(132–181)	46(33–59)	14(7–22)	144(120–167)	45(32–59)

## Discussion

This study documents comprehensive, age-stratified population-based hospitalization and mortality rates associated with AGE and RVAGE before introduction of RV vaccines among Kenyan children < 5 years in a rural community whose demographic and healthcare seeking characteristics are well described [16, 19]. Unlike other WHO rotavirus surveillance study sites in Africa, our hospital-based surveillance site for rotavirus is unique for a few reasons. First, it is supported by an ongoing HDSS which monitors population denominators and conducts verbal autopsy [16]. Second, our surveillance hospital is the only regional public referral hospital for the local HDSS and the only in-patient facility within the HDSS, making our surveillance data representative of the population as shown from our current data and as previously observed [15]. Furthermore, the advantages of a population-based incidence rate are two-fold. First, they provide an opportunity to estimate number of people affected by a disease. Second, they can help to project the number of illness episodes that can be prevented with effectively known interventions such as vaccines [25].

Our 4-year study’s most important findings are that before RV vaccine introduction in Kenya; approximately 90 and 60% of RVAGE hospitalized children were aged < 2 years and < 1 year, respectively, and that hospitalizations and mortality associated with AGE and RVAGE were highest among infants. Furthermore our data suggests that children bearing the greatest burden of morbidity and mortality associated with AGE and RVAGE were infants aged 6–11 months. This finding is similar to observations from neighboring Sudan where pre-RV vaccine data indicates that 91 and 61% of rotavirus hospitalizations occurred before 2 years and 1 year respectively [26]. Furthermore, our finding is consistent with observations from the first 2 years of the current study [15], a study conducted at the coastal region of Kenya [27], Global Enteric Multicenter Study (GEMS) [2] and other studies conducted in Europe [28] before introduction of RV vaccines. Our observation that 21% of hospitalizations among children < 5 years in the HDSS were due to AGE is similar to 23% reported previously from mid-term analysis of our current study [15], 22% reported from Kilifi HDSS in coastal region of Kenya [27], 21% from neighboring Mwanza region in Tanzania [29], and 21% from Ethiopia [30]. In addition, our finding that 26% of hospitalized AGE case patients were infected with RV remains similar to the rate of 27% reported from mid-term analysis of our current surveillance data [15] and to 29% from Kilifi HDSS at the Kenyan coast [27]. These observations suggest that AGE and RVAGE burden in our setting is comparable to those from other settings in Kenya and neighboring countries before RV vaccine introduction. Our observation that rates of hospitalization due AGE and RVAGE declined over the study period before vaccine introduction may be associated with unknown non-RV vaccine intervention factors. However, the proportion of all deaths that were associated with AGE and RVAGE did not follow the same pattern. Thus, these observed trends are difficult to explain, though in part may reflect the effects of other interventions. Although widespread distribution and use of zinc and ORS as part of devolved government development efforts in Kenya has been described [31] and may be a contributing factor to the observed decline in diarrhea burden in this setting, such argument remains speculative and prompts further investigation. This trend however is consistent with other observations from a recent community-based survey conducted in this setting [19], and is not dissimilar to the global trend of diarrhoea and rotavirus disease [3, 32].

Our data show that rotavirus was more commonly detected among infants. Moreover, RVAGE presented with more severe episodes than non-RVAGE as characterized by severe dehydration, vomiting and low grade fever —an observation similar to other previous studies [30, 33–35]. Rotavirus is the most common cause of severe dehydrating diarrhoea and is the leading pathogen associated with moderate-to-severe diarrhoea (MSD) [35], as further reaffirmed by GEMS — the largest diarrhoea etiology case-control study ever conducted in countries representing the highest disease burden regions located in Africa and Asia [2]. Severe dehydration caused by diarrhoea in children is a major cause of preventable morbidity and mortality in Kenya [31]. As commonly observed in our setting and consistent with the caretakers healthcare seeking trends in Kenya [19, 31], delay in seeking care for childhood diarrhoea and reducing amount of fluid and food intake during childhood AGE illness can lead to severe disease. Our current study found that case-fatality among RVAGE was not significantly different from non-RVAGE cases, suggesting that rotavirus may not be associated with mortality in hospital based studies as shown from other studies [33, 36]. This finding supports the assumption that seeking care for RVAGE from a health care facility enables access to appropriate rehydration, which would then reduce the risk of death from the disease.

Understanding seasonality of rotavirus can help formulate hypothesis for assessing potential factors influencing transmission and guide policy makers in deciding on appropriate interventions and approaches that can work in local settings for improving case management during peak seasons [37]. For example, in settings such as USA, rotavirus seasons have been observed to be delayed, shortened, and diminished [4, 38] after vaccine introduction. In our current analysis, rotavirus detection peaked in months which are locally known to be usually warm and dry. Our current findings are consistent with recent observations from Kenya [15, 33], and remains similar to findings from other studies conducted in Burkina Faso [37], Peru and Bangladesh [39] before rotavirus vaccine introduction in those settings. Although there is no unifying rotavirus seasonality pattern globally [40], it’s spread by the faecal-oral route remains agreeable [35], and even airborne or droplet transmission has been postulated [41]. The later attribute potentially makes the virus transmission route also to resemble that of other non-enteric respiratory infectious diseases such as measles [42].These observations suggests that a drop in humidity and rainfall combined with dry soil could potentially increase additional chance for transmission through aerial contaminated faecal materials since survival of rotavirus may still be favored in such conditions as described elsewhere [41, 43].

Treating RVAGE is expensive. In Kenya, it has been estimated that rotavirus disease cost the national healthcare system $10.8 million each year, and that a 2-dose rotavirus vaccine (RVV) series can avert ~ 2500 deaths, ~ 6000 hospitalizations and ~ 860,000 clinic visits with a cost saving of $2.1 million annually [4].RV vaccines have been shown to be effective in reducing the hospitalizations and death due to diarrhoea in children and the protective effect potentially lasts through 2nd year of life [1, 44].While the benefit of these vaccines has been documented in other African countries where they were introduced ahead of Kenya, such as in South Africa [45], Rwanda [3], Ghana [12],and Togo [46], population-level benefits of RVV are yet to be demonstrated from Kenya.

A possible limitation of our current study is that many rotavirus-associated fatalities are likely associated with delay in healthcare seeking [5]. Furthermore, VA relies on signs, symptoms and circumstances prior to death to assign cause of death which is subject to misclassification error, and therefore the method as applied in our current study may lead to over or under-estimation of mortality [36]. Our methodology for estimating diarrhoea deaths attributable to rotavirus was based on the following 3 assumptions: (i) that in the absence of treatment, the hospitalized severe cases would not have survived; (ii) the treatment effect on survival of severe diarrhoea is equal for rotavirus and non-rotavirus diarrhoea; and that (iii) the rotavirus attributable fraction of severe diarrhoea observed in the sentinel hospital are generalizable to the source population within each age stratum as already described elsewhere [36]. Maintaining caution when interpreting these estimates is important since we recognize that such assumptions may affect the validity and generalizability of the estimates to the general population. However, since there are currently no reliable data for the direct measurement of the proportion of diarrhoea deaths that are attributable to rotavirus [22, 36] especially in the high disease burden regions located mostly in low-and middle income countries such as in our setting [3], we believe our methodology remains more reasonable, robust and applicable as recommended by WHO [22, 36]. Moreover, our current hospital surveillance data suggests agreeable representation of the source population consistent with previous observations [15].

## Conclusions

This study shows that AGE and RVAGE associated hospitalization and deaths are high in this setting with children aged 6–11 months bearing the greatest burden. These findings support the introduction of a vaccine that would potentially provide protection to young children before the disease peaks at 6–11 months of age in this setting. While improvements in drinking water, sanitation and hygiene can effectively prevent other forms of diarrhoea, such interventions do not adequately prevent the spread of rotavirus, thus leaving vaccines as the best alternative in preventing AGE and RVAGE in settings such as ours [5]. Continued surveillance will be important for measuring the impact of rotavirus vaccine introduction in Kenya.

## Abbreviations

AGE: Acute gastroenteritis
CDC: US Centers for Disease Control and Prevention
CGHR: Center for Global Health Research
EIA: Enzyme immunoassay
GEMS: Global Enteric Multi-Center Study
HDSS: Health Demographic Surveillance System
KEMRI: Kenya Medical Research Institute
MoH: Ministry of Health
PYO: Person-years of observation
RVAGE: Rotavirus AGE
SAS: Statistical Analysis Software
SCRH: Siaya county referral hospital
VA: Verbal autopsy
WHO: World Health Organization
